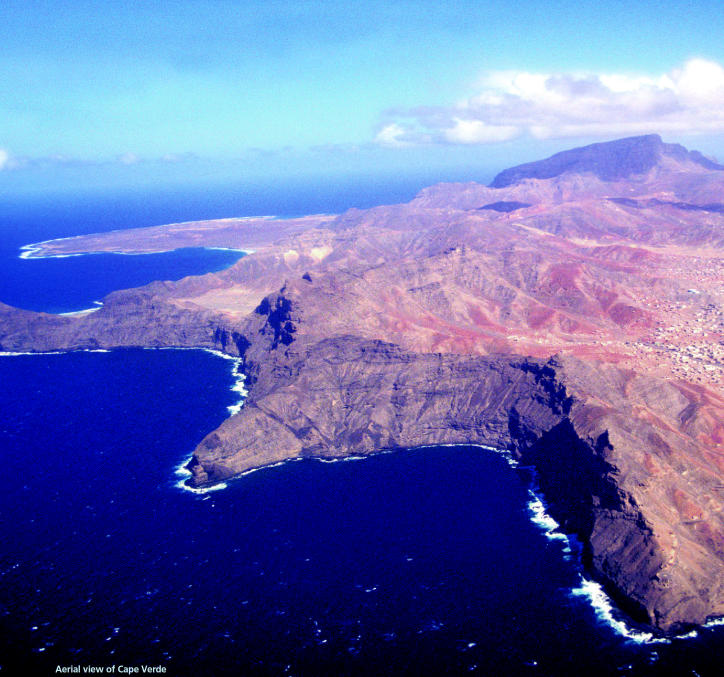# Keeping Afloat: A Strategy for Small Island Nations

**DOI:** 10.1289/ehp.113-a606

**Published:** 2005-09

**Authors:** Charles W. Schmidt

Looking for some adventure? Then how about a trip to Fiji, the Caribbean, or the Seychelles? With their palm trees, lazy beaches, and clear turquoise waters, few places have more appeal than the world’s island nations. Roughly 40 such countries, some sovereign and others not, lie scattered across the planet, ranging in size from Papua New Guinea, with a land area of 452,860 square kilometers and more than 5 million citizens, to Tokelau, a tiny, self-administering territory of New Zealand with a land mass measuring just 10 square kilometers and a population of 1,400.

Perhaps surprisingly, there is no clear official definition for what constitutes an island nation. The Alliance of Small Island States (AOSIS), an ad hoc lobbying and negotiation organization that coordinates the most prominent grouping, refers to its 45 members as “small island developing states,” or SIDS. The criteria that define SIDS are somewhat ambiguous, however. AOSIS members include Belize, Guinea-Bissau, Guyana, and Suriname, which are all coastal—although not technically island—nations. The upper population limit for SIDS, which is 10 million, also appears flexible: AOSIS counts Cuba as a member, despite the country’s population of 11.3 million. AOSIS has even received an application from Madagascar, despite that country’s population of 18 million.

Island nations may be beautiful, but their isolation makes them vulnerable to outside forces that increasingly threaten their survival. Rising sea levels linked to global warming could submerge some altogether. Tuvalu, a West Pacific nation whose peak height rises just 5 meters over sea level, could be uninhabitable within 50 years, some experts say. A similar fate could also doom the Maldives, the Marshall Islands, Kiribati, and Tokelau.

Meanwhile, for reasons not entirely understood, hurricane activity in the tropics appears to be increasing. Hurricane Ivan—the sixth most intense Atlantic hurricane on record—blasted the Caribbean with 160-mile-per-hour winds in 2004, devastating most of Grenada’s housing and virtually destroying its economy within just a few hours. The 2005 Caribbean hurricane season made headlines early on with Dennis in July. This earliest powerful hurricane ever recorded in the region caused an estimated US$5–9 billion in damage.

Most island nations struggle daily with depleted fish stocks, inadequate waste management, ship pollution, degraded reefs, dwindling freshwater supplies, and poverty. In general, the farther an island nation is from global markets, the poorer it is. Distance makes everything more expensive; oil prices in particular tend to be extremely high owing to transportation costs. The more remote islands also tend to lack communications infrastructure, access to information technology, and adequate numbers of trained professionals, including engineers, doctors, and teachers. These technical limitations slow economic development and exacerbate environmental problems caused by poor management of island resources.

Island nations are also uniquely threatened by price shocks from economic globalization. Most island nations depend heavily on tourism, foreign aid, and a limited range of exports like sugar and bananas. Declines in any of these sectors can decimate revenue streams, exacerbating the poverty that already plagues these countries. In St. Lucia, export revenue from bananas plunged from US$46 million in 1996 to US$22 million in 2002, largely because cheaper bananas from Central America flooded world markets during that same period. According to Kishan Kumarsingh, technical coordinator at the Environmental Management Authority in Trinidad and Tobago, climate change can also alter growing conditions, affecting agricultural productivity.

Price shocks emanate in part from the World Trade Organization’s (WTO) trade liberalization policies, which are dismantling arrangements that traditionally have guaranteed markets for island exports. A preferential trade link between the European Union and African, Caribbean, and Pacific Island exporters known as the EU–ACP Agreement is being phased out by the WTO after 30 years in existence as part of a deliberate effort coordinated by the organization to reduce protectionism and open economies to more globalized trade. Its elimination already hurts Caribbean banana exports, as indicated by St. Lucia’s experience. Sugar exports from Pacific countries could suffer similar fates, says François Coutu, a spokesperson in the United Nations (UN) Department of Public Information.

With environmental and economic problems mounting, island nations are turning increasingly to donor countries for aid. But these sources, too, are dwindling: donor nations’ development aid to island nations decreased from a high of US$2.3 billion in 1995 to US$1.7 billion in 2005, according to the UN Small Island Development Unit.

## The Rising Sea

Of all the threats facing island nations, the rise in sea level could be the most catastrophic. Traditionally, sea level was measured with tide gauges including simple graduated measuring staffs and more complex devices that produce continuous graphic descriptions of tide height over time. But in the early 1990s, satellites began generating more comprehensive profiles of global sea level. Thanks to these orbiting systems, scientists now know that the average global rate of sea level rise has increased 50% during the last 12 years—up to 3 millimeters per year from a 50-year annual average of 2 millimeters, according to the National Aeronautics and Space Administration (NASA). Experts attribute rising sea level to global warming and its influence on two key parameters: ocean warming (which causes water to expand) and glacial melting (which is discharging increasing amounts of freshwater directly into the sea).

According to Kevin Trenberth, who heads the Climate Analysis Section at the National Center for Atmospheric Research, the global mean sea surface temperature has risen by roughly 1.5°F since the beginning of the twentieth century. Of that increase, roughly half has occurred since 1970, reflecting the growing effects of global warming from human-induced climate change, he says. The increase in sea surface temperature causes oceans to expand. This thermal expansion, Trenberth says, produces a roughly 1.6-millimeter global rise in sea level every year.

Melting glaciers account for most of the residual rise in sea level, Trenberth says. In a worst-case scenario, enormous ice sheets in Greenland and West Antarctica could melt and raise sea levels by up to 1 meter, suggests Waleed Abdalati, who heads the Cryospheric Sciences Branch at NASA’s Goddard Space Flight Center. Scientists emphasize there currently aren’t enough data to predict when—or even whether—such a scenario might occur. But if it does, many island nations and coastal communities would be inundated: “It’s estimated that more than a hundred million lives are potentially impacted by a one-meter increase in sea level,” Abdalati says.

In the meantime, even the comparatively small increases in sea level seen today can produce large effects, particularly when superimposed on high tides and storm surges, says Laury Miller, who heads NASA’s Satellite Altimetry Laboratory. Miller adds that as a first impact, rising seas contaminate freshwater resources.

This isn’t news to Enele Sosene Sopoaga, AOSIS vice chair and Tuvalu’s UN ambassador. While his country’s water storage capacity has improved, Sopoaga says, the freshwater tables just beneath Tuvalu’s atolls have become brackish and poisonous to root crops including pulaka, a yam-like vegetable that was once a dietary staple. Sea level rise is a constant presence in Tuvalu, he says. Waves crashing just a meter from the main road bring rocks and debris, slowing traffic and endangering lives. And many homes experience flooding whenever tides are high. “Our island is sinking together with our hearts,” he says.

Tuvalu is also battered by tropical storms of increasing ferocity, Sopoaga says, a view supported by new research from the Massachusetts Institute of Technology indicating that hurricanes in both the Atlantic and Pacific oceans have become 50% stronger during the last 50 years. These findings, produced by professor Kerry Emanuel of the Department of Earth, Atmospheric, and Planetary Sciences, appear in the 31 July 2005 online edition of *Nature*.

According to Trenberth, the main factor driving storm intensity is sea surface temperature. Higher sea surface temperatures raise the water vapor retention capacity of the lower atmosphere, which increases rainfall during hurricanes and other tropical storms. Because sea surface temperatures are rising with global warming, more intense weather events may be a direct consequence of human activity, Trenberth says.

Citing their vulnerability to climate change, island nations have long championed the Kyoto Protocol, which seeks mandated reductions in the greenhouse gases linked to global warming. But Tom Wigley, a senior scientists at the National Center for Atmospheric Research, says the greenhouse gases in the atmosphere now will likely linger for up to 100 years. Even if future emissions were limited to current levels, greenhouse gases would continue to rise and pose ongoing threats, he says. This is due to the lag-time response of the atmospheric and climate system—the climate does not react immediately or in the short term to greenhouse gas emissions, but rather over long periods.

## Adapting to Climate Change

Small island nations faced with the consequences of climate change must somehow adapt to it. Only a few options are available, however, and none of them are attractive. According to Kumarsingh, islanders can either abandon threatened areas, retreat to higher ground, or build walls to hold back the sea. The Maldivian capital of Male is partially ringed by a system of protective walls that was built at a cost of US$4,000 per meter, financed largely by Japan. These walls saved the capital from the great tsunami that struck the Indian Ocean in December 2004. But seawalls are not always so effective; according to Sopoaga, most built in Tuvalu are damaged and in urgent need of repair.

Kumarsingh says coastal retreat could be highly challenging. “It’s not so simple,” he explains. “You have to relocate communities, amenities, and services, which is expensive. Island cultures are rooted in coastal living, so relocation has many socioeconomic impacts.”

Further, he adds, some islands, such as the Maldives, do not have higher ground to which to retreat. The size of many islands also limits how far people can go to escape: “When you retreat from one coast, chances are you may end up on the other coast. So where do you go?”

Perhaps most importantly, these strategies address only one aspect—sea level rise—of the consequences of global warming. Among the other direct and indirect consequences are changes in agriculture and food production, biodiversity loss, damage to coastal coral reefs (which are sensitive to warm sea temperatures and which act as natural coastal defense structures by dampening wave energy), saltwater intrusion to coastal aquifers (making potable water production more expensive), and increases in certain disease vectors due to increased humidity.

## The Mauritius Strategy

Today, island nations are trying to promote global awareness of their plight. Earlier this year, the world spotlight shone on island nations during a large international meeting held in Port Louis, Mauritius. The January meeting, sponsored by the UN, brought together 2,000 delegates and numerous heads of state to review progress on a 10-year action plan for island nations called the Barbados Programme of Action (BPOA). Stakeholders acknowledged that the BPOA—which since 1994 has sought to improve environmental management and economic development in island nations—has fallen far short of its goals. One UN official attributed the BPOA’s failures in part to bureaucratic inertia. “The UN never set up an appropriately sized implementation office, and we had problems with no new funds being pledged [by donor countries],” the official explains. “Furthermore, the [island nations] never made the plan a cornerstone of their aid dealings.”

Hoping to redress these shortcomings, delegates pushed forward by drafting a new plan called the Mauritius Strategy. “The Mauritius Strategy has clear recommendations that if taken seriously will make up for the deficiencies of the last ten years,” says Anwarul K. Chowdhury, under-secretary-general for the High Representative for the Least Developed Countries, Landlocked Developing Countries, and Small Island Developing States (UN-OHRLLS). The 30-page strategy lays out a detailed agenda focused on issues such as climate change, sea level rise, natural disasters, waste management, water resources, energy, technology, sustainable development, and tourism.

Stakeholders hope the renewed momentum will fuel concrete progress. According to Om Pradhan, chief of the UN-OHRLLS Policy Development and Coordination Monitoring and Reporting Unit, international delegates appeared receptive to the view that island nations face unique challenges. The European Union in particular, he says, was forthcoming in its support for the islands—which is critical because European countries provide aid and comprise key markets for island exports.

Responsibility for the strategy’s implementation falls to the UN Department of Economic and Social Affairs (UN-DESA) and the UN-OHRLLS, among other divisions. As such, the UN will help island nations prepare to claim that they deserve special attention from the international community, says Diane Quarless, who heads the UN-DESA Small Island Developing States Unit.

But these claims could be met with some resistance, she admits. This is in part because some island nations are relatively well off: Singapore and Aruba, for instance, each boast per-capita incomes of US$28,000, values that in Quarless’s opinion, are skewed high by the wealth of a minority. “The international community looks at per-capita income, and says, ‘These guys are rich, and they live in paradise. Why should we give development assistance to them?’” Quarless says. “But we say that it’s not all paradise in the island nations. Economic and environmental vulnerability are universal across the board—even the richer countries could be wiped out by a single natural disaster. The international community puts an inordinate weight on income; we want them to emphasize environmental vulnerability when they decide if island nations are worthy of preferential treatment.”

A key objective, Quarless says, is to convince the UN General Assembly that environmental vulnerability—not income—should determine island eligibility for developing country assistance. As it stands now, the UN selects nations for its list of Least Developed Countries (LDCs) on the basis of income. LDCs are eligible for special concessions, such as access to duty-free export markets and high levels of developmental aid. Several island nations—including the Maldives, Cape Verde, and Samoa—may soon be “graduated” from this list because of their rising per-capita income. Quarless argues that, because they face extraordinary environmental challenges, these countries should be allowed to retain their LDC status. “A country like Maldives, which is quite literally under threat of disappearance, should not have to face the challenge of fending for itself,” she says.

Additional UN advocacy on behalf of island nations now targets institutions including the World Bank and the WTO, Pradhan adds. These institutions don’t currently accept “islandness” as a special category, he says. “This is something we’re trying to change in view of [island nations’] greater vulnerabilities to social, economic, and environmental shocks,” he says.

Inasmuch as island nations need global support, however, they must also promote their own sustainable development, making the most productive use of island resources. Stakeholders in Fiji, for instance, have recently developed automotive fuels using coconut oil.

Lelei LeLaulu, president of Counterpart International, a U.S.–based nonprofit company that promotes sustainable development projects around the world, argues that island nations need to emphasize sustainable tourism as a cornerstone of development. With this approach, island nations recognize that resource attractions—for instance, coral reefs—can’t be protected in isolation from their larger ecosystems. Conservation should address large natural areas rather than small pockets with tourist appeal, such as specific beaches, LeLaulu says. Moreover, he adds, island nations should strive for greater community ownership of tourist enterprises to ensure that natural resources are available for future generations. With a greater stake in tourism, islanders may do more to prevent coastal pollution and to regenerate the coral reefs that attract visitors and sustain local ecosystems.

Other experts point out that economic diversification is also essential for islands that depend heavily on tourism. “The traditional generic island tourism product that readily attracts foreign earnings—sand, sea, coastal hotels, and so forth—are the very amenities that are under threat from sea level rise and tropical cyclones. . . . The challenge here is the development of a tourism product that is attractive outside of the traditional attractions,” says Kumarsingh. He argues that economic diversification is essential for those economies that are heavily dependent on tourism. “Otherwise,” he says, “[island nations] may be setting up themselves for a greater fall—putting all their eggs in a very vulnerable basket.”

Ultimately, island nations are world treasures whose health may portend the future of the planet. Both their inhabitants and the millions who come visiting every year have a stake in their survival. Postcards sent today depict peaceful serenity, but behind these nations’ attractive façades lie many difficult challenges. As the waters and the pressures rise it remains to be seen whether the world’s island nations will sink or swim.

## Figures and Tables

**Figure:**